# Health related quality of life of children and adolescents with congenital adrenal hyperplasia in Brazil

**DOI:** 10.1186/s12955-014-0107-2

**Published:** 2014-08-13

**Authors:** Daniel Luis Schueftan Gilban, Paulo Alonso Garcia Alves Junior, Izabel Calland Ricarte Beserra

**Affiliations:** Instituto de Puericultura e Pediatria Martagão Gesteira, Universidade Federal do Rio de Janeiro, Rio de Janeiro, Brazil; Rua Tenente Marones de Gusmao, 23/205, Copacabana, Rio de Janeiro, RJ 22041-060 Brazil; Rua Paulo Silva Junior, 201/909, Méier, Rio de Janeiro, RJ 20735-230 Brazil; Praça Tele Santana 45, bloco 2, ap 302, Barra da Tijuca, Rio de Janeiro, RJ 22793-298 Brazil

**Keywords:** Congenital adrenal hyperplasia, Quality of life, Glucocorticoid, Children and adolescents

## Abstract

**Background:**

Congenital Adrenal Hyperplasia (CAH) is an endocrine disorder characterized by enzymatic deficiency in adrenal steroidogenesis, leading to adrenal insufficiency and hyperandrogenism. Patients need continuous hormone replacement therapy, but adequate control has proven challenging, exposing patients to undesirable consequences of both disease and treatment.

**Objective:**

To evaluate the health related quality of life (HRQoL) of children and adolescents with CAH due to 21-hydroxylase deficiency.

**Methods:**

In an analytical study, generic questionnaires, validated and translated versions, *Pediatric Quality of Life Inventory 4.0* (for self-assessment of patients) and *Child Health Questionnaire - PF50* (for parents) were applied and mean scores were analyzed with Student’s t-test.

**Results:**

We included 25 patients (19 female) with classical CAH (14 salt wasting/11 simple virilizing), mean age ± standard deviation (SD) of 11.4 ± 3.6 years (5–17.9), and their parents. Self-assessment of HRQOL showed decrease in mean scores: overall (67.8 ± 15.5 vs. 88.9 ± 7.4, p value = 0.015) and in the physical (75.2 ± 15.0 vs. 95.9 ± 5.8, p value = 0.014) and psychosocial (63.9 ± 17.8 vs. 85.0 ± 9.6, p value = 0.023) dimensions of patients, compared to healthy controls (previously published national data on children and adolescents). The assessment of the parent’s view was concordant, also showing losses in the physical (43.7 ± 8.0 vs. 55.1 ± 3.6, p value = 0.013) and psychosocial (41.9 ± 9.7 vs. 53.0 ± 7.0, p value = 0.025) dimensions. The comparison of HRQOL between subgroups 1) males versus females and 2) salt-wasting versus simple virilizing showed no significant differences.

**Conclusion:**

There seems to be a loss of HRQOL in children and adolescents with classical CAH. The self-assessment was concordant in key areas with the assessment made by their parents. No differences were observed between genders or clinical presentation of the disease.

## Background

CAH is the most common endocrine disorder of genetic origin, characterized by specific enzyme deficiency that leads to defects in adrenal steroidogenesis, resulting in adrenal insufficiency and hyperandrogenism [1].

In 95% of cases, mutation occurs in the CYP21A2 gene, causing varying degrees of inactivity of the 21-hydroxylase enzyme, with consequent defects in the final products of the glucocorticoid (GC) and mineralocorticoid strains. This inability signals to the hypothalamus-pituitary axis the elevation of adrenocorticotropic hormone (ACTH), but the greater stimulation of adrenal eventually leads to excess of androgenic precursors, since there is no interruption in this hormonal lineage [1].

The clinical expression of CAH depends on the degree of residual enzyme activity, and correlates with the genotype of the patient [1,2]. The classic form of the disease shows signs and symptoms at birth or in the early months of life. In this group of patients, 75% have combined deficiency in the formation of GC and mineralocorticoid, called Salt Wasting (SW) form. This is the most severe presentation of the disease and can be predicted by genotype “null” and “I2splice” [3]. Clinical manifestations include adrenal insufficiency because of GC deficiency and adrenal crisis because of salt loss leading to dehydration, shock and hyponatremia. These are the most feared manifestations due to high potential for morbidity and mortality [4].

The remaining 25% are Simple Virilizers (SV), where the reduced enzymatic activity is adequate in most cases to prevent deficiency of mineralocorticoid, thus preventing adrenal crisis due to salt-loss. GC deficiency is present, but with less exuberant symptoms [2].

All patients with SW and SV forms also have hyperandrogenism, starting early in fetal life. Male patients present few clinical manifestations at birth, as increased penile length and hyperpigmentation of the scrotum are not easily recognizable. However, female patients affected present varying degrees of virilization of the external genitalia, featuring a disorder of sex development (DSD) with ambiguous genitalia. CAH is the leading cause of DSD [5].

Patients have a chronic and life-threatening condition, and therefore need medical attention and continuous treatment. The treatment is based on GC replacement, in a dose sufficient to cause suppression of ACTH levels and decrease hyperandrogenism. Although simple in principle, in practice the treatment has been a great challenge and a difficult balance between hyperandrogenism and hypercortisolism, since hormonal replacement therapy is imperfect and does not mimic the physiological secretion of these hormones [4]. It is observed that the dose of GC required for suppression of ACTH levels is supraphysiological [6], and therefore poses risks of adverse effects related to hypercortisolism.

Female patients with genital ambiguity usually undergo feminizing surgery in order to align the external genitalia to the assigned gender, as the clinical treatment prevents the progression but does not reverse virilization that has occurred prenatally.

The importance of psychological support to patients with DSD and specially with CAH, as well as their families, is emphasized in the various consensus of many experts in the field [1,4,5] and is an important part of the treatment, although often underestimated.

The term quality of life (QOL) is difficult to define, but with a deep inner meaning of overall satisfaction with life, in the physical, psychological, social and spiritual dimensions [7]. The World Health Organization defined it as “the individual’s perception of their position in life, in the context of culture and value systems in which they live and in relation to their goals, expectations, standards and concerns” [8].

However QOL is influenced by a variety of conditions that can affect an individual’s perception, feelings and behaviors in their daily functioning, including their health condition. Likewise, promoting health doesn’t necessarily promote QOL, but one of the many items that comprises it. Thus, a new concept arises, the health related quality of life (HRQoL), facing the repercussions that the current state of health of the individual can have on his overall satisfaction with life [9].

Chronic diseases affect 18% of children and adolescents with high survival rates (90% beyond the second decade of life) [10]. Many require daily self-care and health management that may restrict physical and social activities [10,11]. Sick children are even more likely to have psychological consequences, which may be affected by therapeutic modalities [11]. HRQOL as an indicator has been increasingly used as a supplement to biological or clinical measures of disease, ensuring that the patients’ perspective on the disease, the need for care and their preferences for treatment modalities are taken into account [12].

CAH could directly impact HRQOL on the following conditions: genital ambiguity; impaired growth; higher incidence of obesity, metabolic syndrome and hypertension; acute adrenal crisis; losses in psychosexual milestones. However there is little information available about how the condition affects the HRQOL of these patients.

## Objective

To evaluate the HRQoL of children and adolescents in Brazil with classical CAH due to 21-hydroxylase deficiency and the perceptions of their parents in the various dimensions that comprise QOL, taking into account the gender of the patient and presentation form of the disease.

## Patients and methods

We included 25 patients with classic CAH due to 21-hydroxylase deficiency followed in the endocrinology department (14 SW and 11 SV), mean age ± standard deviation (SD) of 11.4 ± 3.6 years (5–17.9), whose parents and/or guardians were literate. This represents all of the patients followed between September 2011 to September 2012 who met age criteria (5 to 18 years old), since there was no refusal to participate. The study was performed in a University Pediatric Hospital setting, approved by the Research Ethics Committee and parents and/or guardians signed informed consent. All patients had a clinical diagnosis based on signs and symptoms and biochemical diagnosis based on 17-hydroxyprogesterone levels. No molecular analysis was performed.

The control group consisted of healthy individuals from the instruments’ previously published validation studies for the Brazilian population of children and adolescents [13,14].

Two instruments were used to evaluate the HRQoL: Child Health Questionnaire - PF50 (CHQ) for the parents’ perspective and Pediatric Quality of Life Inventory 4.0 (PedsQol 4.0) for patients. The choice of these instruments was based on the following conditions or characteristics: they were widely used in previous studies that assessed HRQOL in children and adolescents; have been translated and validated for use in the Brazilian population; have desirable characteristics for evaluation of HRQOL as: good psychometric properties; ease to comprehend and to apply; they are self-administered, ensuring the subjectivity of responses; they assess multiple aspects of QOL, ensuring the multidimensionality of the construct; have standard values for the Brazilian population of healthy children and adolescents; PedsQol 4.0 presents different versions for age groups (5–7, 8–12 and 13–18 years), respecting the cognitive development of children; and the absence of a specific questionnaire validated for the Brazilian population.

The CHQ questionnaire addresses multiple domains of physical, mental health and functionality. The instrument was designed for use in parents of children 5–18 years old, healthy or with chronic diseases, and analyzes the physical and psychosocial domains through structured questions with answer options. The questionnaire consists of 50 questions comprising 15 scales. The evaluation of each question uses the method of added points - Likert method. The final score of each scale ranges from 0 to 100. Ten scales are used to compose the aggregate scores that summarize the assessment of physical and psychosocial functions, with scores from 0 to 50 for each aggregate score and five scales are evaluated individually. The score calculation was based on the orientation guide of the questionnaire.

The cultural validation study for the Brazilian population of the CHQ was made to the form applicable to parents/guardians and used 314 healthy individuals [14]. This instrument was used in our study as self-administered to parents/guardians, as a way to assess the perceptions of caregivers and compare them to the perception of the carriers of CAH.

The generic questionnaire PedsQol 4.0 was designed for modular assessment of HRQoL by self-administration in children and adolescents between 5 and 18 years of age. Presents 23 items covering : physical functioning (eight items), emotional dimension (five items), social dimension (five items) and scholar dimension (five items), ensuring the attributes of subjectivity and multidimensionality. The questionnaire has three different age groups versions: 5–7 years, 8–12 years and 13–18 years. The items for each of the forms are essentially similar, differing only in terms of appropriate language for each stage of cognitive development. Response options are scored according to the user manual provided and implemented on a scoring scale from 0 to 100 points, with higher scores meaning better HRQOL. The aggregate score for physical health scale corresponds to the physical dimension (8 items). The aggregate score of psychosocial health is the average computed as the sum of items answered on scales of emotional, social and educational dimensions divided by the number of items.

The cultural validation study of the PedsQol 4.0 for use in Brazilian children and adolescents used 180 healthy individuals, corresponding to the standard of the healthy population as control group in this study [13]. This study confirmed the feasibility, reliability and validity of the Brazilian version 4.0 PedsQol. This version also had good correlation with the Brazilian version of the CHQ (p value < 0.005) [13].

The questionnaires were conducted by the principal investigator, according to the instruments’ guidance manuals. Patients and parents recruitment occurred in the waiting room of a pediatric endocrinology outpatient clinic before the routine consultation, in order to prevent that medical opinion could somehow influence the perception of patients. Only one parent or guardian completed the questionnaire.

Printed forms of questionnaires were used. Self-administration was strictly respected in a private office. For children 5–7 years, the items were read aloud by the investigator, as well as answer options. Although in the same room, children and parents could not opine or influence each other’s responses. For questions on any item, investigator merely read aloud the item without changing the wording of the phrase in printed form. The answer options were converted into a rating scale from 0 to 100 points (provided in the user manual) where higher scores mean better HRQOL.

Data were analyzed using Epi Info 6. Results were expressed as mean ± SD. We used Student’s t test for comparison between means of independent samples and considered statistically significant p value < 0.05. The variables were verified for Kolmogorov-Smirnov test and normal distribution hypothesis was not rejected according to histogram plotting.

## Results

### Characteristics of the sample

No patient or parent denied participation in the study. Of the 25 patients, with mean age ± standard deviation (SD) of 11.4 ± 3.6 years (5–17.9), 6 were male and 19 female, but among the 19 female patients, 2 opted for male social sex. Among the girls, 3 were assigned as boys at birth, requiring change of civil registration (within the first year of life).

Age at diagnosis ranged from 9 days to 7.58 years, mean ± SD of 0.1 ± 0.04 in the SW and 2.0 ± 2.5 years in SV, respectively. No patient was diagnosed by newborn screening. Twelve of 14 SW patients had acute adrenal crisis at diagnosis, the other two patients had subsequent adrenal crisis.

Only 3 patients were treated with hydrocortisone. The others used Prednisolone (n = 14), Prednisone (n = 5) or Dexamethasone (n = 2). One patient, at the time of the study, was not in use of GC because of poor adherence and treatment dropout, but spontaneously returned to attendance. Based on clinical descriptions from medical records and opinions of medical staff that often consult those patients, adherence to treatment recommendations was considered good in 11 patients, regular or questionable (with conflicting information) in 7 patients, and poor in 7 patients, of whom 3 (patients 1, 6 and 20) were without regular clinical follow up for a long period of time (>12 months), but spontaneously returned to attendance.

Considering the single dosage hormone levels (serum androstenedione and renin) up to 120 days prior to study participation, in 10 patients control was good (levels within reference range), in 6 regular (levels up to twice the upper limit of normal) and in 9 bad (levels markedly altered). On average, bone age was 2.8 ± 2.2 years ahead compared to the chronological age.

Table 1 summarizes the main characteristics of the sample.

**Table 1:** **Clinical characteristics of the sample**

**Patient (N°)**	**Age (years)**	**Sex**	**Sex (rearing)**	**Clinical presentation**	**Age at diagnosis (years)**	**GC in use**	**Adherence at therapy**
1	17.9	F	M	SV	0.6	Prednisone	Bad
2	7.5	F	F	SW	0.2	Hydrocortisone	Good
3	7.9	F	F	SV	0.6	Prednisone	Regular
4	14.4	F	F	SW	0.2	Prednisolone	Bad
5	16.4	F	F	SW	0.1	Prednisolone	Regular
6	9.6	M	M	SW	0.1	-	Bad
7	15.4	F	F	SW	0.1	Dexamethasone	Good
8	17.9	M	M	SW	0.1	Prednisone	Good
9	12.4	F	F	SV	0.2	Prednisolone	Good
10	8.1	F	F	SV	0.1	Prednisolone	Good
11	12.0	F	F	SV	3.0	Prednisolone	Regular
12	13.2	M	M	SV	5.0	Prednisolone	Regular
13	7.5	F	F	SW	0.1	Hydrocortisone	Good
14	7.2	F	F	SW	0.1	Prednisolone	Good
15	11.3	F	F	SW	0.1	Prednisolone	Equivocal
16	11.7	F	F	SV	7.5	Prednisolone	Bad
17	14.2	F	F	SV	0.5	Dexamethasone	Bad
18	5.0	F	F	SW	0.1	Hydrocortisone	Good
19	12.8	M	M	SW	0.1	Prednisolone	Equivocal
20	15.8	F	M	SV	1.0	Prednisone	Bad
21	10.9	F	F	SV	0.3	Prednisolone	Good
22	6.4	M	M	SW	0.2	Prednisolone	Bad
23	13.4	F	F	SW	0.1	Prednisone	Equivocal
24	9.6	F	F	SW	0.1	Prednisolone	Good
25	8.4	M	M	SV	5.6	Prednisolone	Good

F: female, M: male, GC: glucocorticoid.

### Self-evaluated HRQoL by the patients

Self-assessment of HRQOL using the instrument PedsQol showed significant decrease (p value = 0.015) of the mean values of the total score of HRQoL of patients (67.8 ± 15.5) compared to healthy controls (88.9 ± 7.4). When evaluated separately, the dimensions that comprise HRQL also showed reduction in mean scores of patients compared to healthy controls.

In the physical dimension, the mean score of the patients (75.2 ± 15.0) was significantly lower than the mean score of the control group (95.9 ± 5.8), the same occurring in academic dimension.

For emotional and social dimensions reductions in mean scores of patients compared to healthy controls were 57.6 ± 23.2 vs. 73.0 ± 16.5 and 76.6 ± 21.3 vs. 93.1 ± 10.5, respectively, without statistical significance (p value = 0.084 and 0.061). However, when considering the psychosocial dimension, which combines the emotional, social and academic dimensions on an aggregate score, patients had a mean of 63.9 ± 17.8, significantly lower than the mean of healthy controls, 85.0 ± 9.6. These results are shown in Table 2.

**Table 2:** **HRQoL assessed by patients**

**PedsQol**	**Patients CAH (n = 25)**	**Controls (n = 180)**	**p value**
**Dimensions**	**(Mean ± SD)**	**(Mean ± SD)**	
Total	67.8 ± 15.5	88.9 ± 7.4	0.015
Physical	75.2 ± 15.0	95.9 ± 5.8	0.014
Emotional	57.6 ± 23.2	73.0 ± 16.5	0.084
Social	76.6 ± 21.3	93.1 ± 10.5	0.061
Academic	57.6 ± 23.0	89.3 ± 11.8	0.015
Psychosocial	63.9 ± 17.8	85.0 ± 9.6	0.023

### HRQOL assessed by the parents

The assessment of HRQOL of children and adolescents with CAH by their parents through the instrument CHQ also showed reduction in mean scores in all dimensions evaluated, when compared to healthy controls.

The aggregate score of physical health of patients had a mean of 43.7 ± 8.0 versus 55.1 ± 3.6 in the control group (p value = 0.013). The same happened to the aggregate score of psychosocial health: 41.9 ± 9.7 in patients and 53.0 ± 7.0 in the control group, with significance.

When analyzed separately, each of the dimensions that make up the aggregate scores also showed lower values in patients, as shown in Table 3.

**Table 3:** **HRQoL scores evaluated by CHQ**

**CHQ Dimensions**	**Patients CAH (n = 25)**	**Controls (n = 314)**	**p value**
	**(Mean ± SD)**	**(Mean ± SD)**	
Aggregate physical	43.7 ± 8.0	55.1 ± 3.6	0.013
Aggregate psychosocial	41.9 ± 9.7	53.0 ± 7.0	0.025
General health	62.4 ± 24.8	92.7 ± 11.6	0.021
Physical functioning	83.0 ± 21.0	98.5 ± 7.9	0.067
Role/social limitations (emotional/behavioral)	77.9 ± 22.7	97.1 ± 9.5	0.051
Role/social limitations (physical)	81.8 ± 19.4	98.0 ± 11.0	0.052
Bodily pain	68.0 ± 22.9	94.2 ± 12.8	0.025
Behaviour	58.0 ± 15.5	79.5 ± 13.9	< 0.0001
Global behaviour	63.4 ± 25.2	85.5 ± 16.1	0.048
Mental health	66.4 ± 19.1	78.2 ± 14.3	0.094
Self-esteem	74.3 ± 22.4	90.3 ± 15,3	0.072
General health perceptions	53.2 ± 18.0	78.3 ± 12.3	0.014
Parental impact (emotional)	49.6 ± 24.5	82.3 ± 21.5	< 0.0001
Parental impact (time)	70.8 ± 26.5	94.2 ± 12.5	0.047
Family activities	77.0 ± 21.7	90.5 ± 13.4	0.092
Family cohesion	65.2 ± 30.4	78.1 ± 19.1	0.16

Although the main point of the instrument is to measure patients’ HRQOL (by the parents’ perspective), two items (emotional and time spent caring for children) also analyzed the impact that the disease causes on parents. In both there was significant difference compared to parents of healthy controls: the emotional impact score of 49.6 ± 24.5 vs. 82.3 ± 21.5 (p value < 0.0001) and the impact of time, 70.8 ± 26.5 vs. 94.2 ± 12.5 (p value = 0.047). Two other items (family activities and family cohesion) evaluated the ability that the family had to absorb the impact of the diagnosis and treatment needs while maintaining their daily routines and living well. These differences were not significantly different when compared to controls.

Finally, the item changes in health, which is not a quantified score, asked parents to compare their children’s health today over a year ago. Fourteen (56%) thought that their children’s health was much better today than a year ago, 5 (20%) thought that there was a slight improvement in their health and 6 (24%) thought that their health was the same as it was a year ago. No parent judged deterioration in health status of their children in the last year.

### HRQOL by gender: male and female

The HRQL scores of patients with sex of rearing male versus female are shown in Table 4. There was no significant difference between groups in the areas assessed. Two patients 46XX (group male) have gender dysphoria and currently live as boys (patients 1 and 20).

**Table 4:** **HRQoL dimensions related to sex of rearing: female versus male**

	**Female (n = 17)**	**Male (n = 8)**	**p value**
	**(Mean ± SD)**	**(Mean ± SD)**	
PedsQol total	69 ± 13.7	65 ± 19.4	0.56
PedsQol physical	75.1 ± 15.6	75.3 ± 14.3	0.97
PedsQol psychosocial	65.8 ± 15	59.7 ± 23.1	0.43
CHQ physical	43.4 ± 8.6	44.3 ± 7.1	0.79
CHQ psychosocial	41.9 ± 10.4	41.7 ± 8.3	0.96

### HRQOL in SW versus SV

The results of HRQoL scores are shown in Table 5. Again, there was no significant difference between the groups, except in the physical dimension of the CHQ. In the parents’ perception, SV patients have greater impairment in physical dimension than SW: 41.1 ± 3.6 vs. 45.7 ± 6.2 (p value = 0.038).

**Table 5:** **HRQoL dimensions in SW versus SV**

	**SW (n = 14)**	**SV (n = 11)**	**p value**
	**(Mean ± SD)**	**(Mean ± SD)**	
PedsQol total	67.3 ± 17.7	68.4 ± 12.8	0.86
PedsQol physical	75.8 ± 14.9	74.3 ± 15.7	0.80
PedsQol psychosocial	62.8 ± 20.5	65.2 ± 14.3	0.74
CHQ physical	45.7 ± 6.2	41.1 ± 3.6	0.038
CHQ psychosocial	43.6 ± 9.1	39.6 ± 10.3	0.31

## Discussion

HRQoL is a topic that has been gaining interest in recent decades as an indicator and predictor of therapeutic outcomes in chronic patients. CAH, on account of the clinical features mentioned and the obvious difficulties in living with a disease requiring continuous administration of GC, may cause losses in HRQoL in patients and their families.

There is no reason to assume that the HRQoL implications are present only in adults. In fact, due to the characteristics of the disease, it is reasonable to assume that children and adolescents with CAH experience significant physical and psychosocial consequences.

Despite the importance of good clinical control and an appropriate surgical management for patients with genital abnormalities, HRQoL of these patients depends largely on the psychosocial management [15], and therefore depends on tools to identify, early in childhood, which patients need the most attention in this regard.

In respect to our sample some points call attention. First, a sample of 25 patients clearly decreased the statistical power of the study, effect partially compensated by large control groups used in this study. Still, some of the findings in this study could be a result of this lack of power. A small sample of patients is relatively common in cross-sectional studies of rare conditions such as CAH, except in multicenter studies, which was not the case in this study. However, we emphasize that our sample is one of the first exclusively composed by pediatric patients with classical CAH.

The female:male ratio of our sample could be explained by the increased mortality of male patients with severe forms of CAH. The absence of genital abnormalities and the gap between discharge from maternity and initiation of symptoms makes those patients more vulnerable to adrenal crisis.

As to the form of treatment, only three patients used hydrocortisone, the first choice for treatment of CAH in childhood. This profile reflects the difficulty in obtaining a formulation of imported source (not available in our country) with consequent higher cost for the study population. Although the socioeconomic profile of our sample has not been evaluated, it is safe to say that many of our patients, enrolled in a public health service and residents of areas composed mainly of low-income families, have significant financial constraints. This reflects a similar profile as the control population group used, that represents national data.

Interesting perspective was offered by Zainuddin and colleagues [16], about the difficulties faced by patients with CAH in developing countries, the reality in many respects close to our sample. In this review some issues were considered, such as poverty, ignorance, lack of basic medical knowledge, lack of psychological therapy and financial difficulties as possible risks factors to HRQoL, reality not experienced by other study populations in developed countries.

Regarding treatment adherence, 11 patients had reported good adherence to treatment guidelines, and only 10 had follow-up tests within normal limits expected. These data support previous reports on the difficulty of achieving good adherence to medical treatment in CAH [17]. In the largest cohort published to date, only 30% of patients with classical CAH had acceptable hormone levels [18].

Self-assessment of HRQoL showed significantly lower scores for physical, psychosocial and total scores when compared to healthy children and adolescents, showing damage caused by the disease or the need for continued treatment. Similar results were shown in previous studies, but not by consensus.

Early work on HRQoL of patients with DSD showed positive results (better than controls) [17,19], or no difference in HRQoL comparing to the general population [20]. Similar results were reported in patients with various chronic diseases, and this data is generally attributed to the ability to generate strategies for dealing with the condition [21]. Only more recent studies have begun to show worse results in HRQoL of patients with DSD, especially in the social dimensions [2,7]. There are also reports of psychiatric morbidity with increased anxiety and suicidal thoughts stated in this population [2].

Johannsen and colleagues [22] evaluated the QoL of 70 adult women with DSD, of which 33 women with CAH (5 with non-classical). They verified a loss on HRQoL of patients with CAH especially, as well as other conditions leading to virilization. However, the instrument used in this work was originally created for use in patients with growth hormone deficiency.

Subsequently, a Swedish study that evaluated only adult women with CAH showed no significant difference in HRQoL scores and wellness [21]. However, spontaneous reports of patients cited fewer chances of marriage, delay in psychosexual milestones and lack of interest in sexual activities. Half of the patients reported that the disease negatively influenced how they were raised and intensified the sense of being different from the social group, especially in those with severe enzyme deficiency. This study showed that the instrument used as objective measure of HRQoL did not match the speech of patients, when analyzed in a qualitative manner, and that this should be a methodological precaution to be taken. In fact, this year an epidemiological study confirmed suboptimal psychosocial outcomes in CAH patients in Sweden, as they had more disability pension and sick leave then general population, besides difficulties in education and carreer management, specially in females [23].

In 2010 a large British multicenter group study (CaHASE) published results on QOL in a cohort of 203 adult patients with CAH [5], including men and patients with non-classical form. They used generic questionnaires and a specific questionnaire (CAH wellbeing) created by the group, showing impairment in all domains of QoL analyzed, although only 23% reported concern about the impact of CAH in their long term health. Reduction in adult height had less impact on the well-being perception than obesity or impaired sexual life. Simultaneously Nermoen evaluated 104 adult patients with classical CAH and found impairment in all domains of QoL [24], with emphasis on general health perception and vitality. In both studies, participation rates were 53 and 76%, respectively. Many refused to answer the questionnaires, which could mean that the HRQoL of patients is further compromised, since in general the no responders tend to have poorer health, may be depressed or socially isolated [16,24].

More recently a study showed that, surprisingly, the HRQoL of patients with CAH is less affected than that of patients with primary adrenal insufficiency [25]. This work led to the hypothesis that hyperandrogenism partly offsets the effects of fatigue observed in adrenal insufficiency, thus improving the well-being of patients. Another issue raised was the difference in the perception of patients with congenital and acquired diseases, with greater psychosocial impact in the latter. Moreover, regarding CAH, some clinical features of hyperandrogenism can actually concern the doctors more than the patients [26].

Two recent reviews [3,27] reaffirmed that the current knowledge on the psychosocial health and well-being in CAH patients remains unclear and consider that the use of different instruments to assess HRQoL do not facilitate comparison between studies. They also emphasize the need for further clarification regarding two issues: at what time of life possible repercussions on HRQoL would be present and the influence of specific sequelae of CAH in this picture. A specific assessment tool validated for CAH patients could contribute to this knowledge.

In 2012, the first study exclusively with children with CAH was published [28]. This multicenter Dutch study investigated the HRQoL of 106 patients, corresponding to 70% of patients who responded to a specific, non validated questionnaire, by mail. However, as a major limitation of the study, the questionnaire was responded by parents with self-administration done only by teenagers. This may have impaired the subjectivity and reliability of responses. Most parents (98%) were satisfied with the overall health status of the children and 88% reported that they have reached the right dose for hormonal control. Most teens (84%) reported no problems in controlling the condition. These results showed that children with CAH suffer few negative effects on HRQoL. Thus, the difference between our results and those of the other sample composed exclusively of children and adolescents with CAH is remarkable. Treatment results between countries due to different socioeconomic background may explain this discrepancy.

Our study evaluated the parents’ perception on the HRQoL of patients with CAH through the instrument CHQ. The results of significantly lower scores in the physical and psychosocial dimensions showed agreement with the views of children and adolescents, assessed by the instrument PedsQol. This result confirms previous data on the good correlation between the Brazilian versions of the instruments [13], in addition to emphasizing the importance of using appropriate instruments to assess HRQoL.

The items of the CHQ that evaluated impact on parents in relation to the time spent in child care and the negative emotional charge of dealing with a chronic illness confirmed that our patients’ parents, when compared to healthy controls, showed a significant negative impact and consequent psychological distress. However, the items that assessed family cohesion showed no significant differences, probably implying that these families develop resilience strategies for a good family life.

To raise children with chronic diseases is difficult, stressful, and can potentially affect the parenting style, resulting in poorer outcomes for patients [29]. Due to the psychological morbidity, parents’ perception about their children’s health may also be negatively affected, beyond the perception of patients themselves [13,30]. Thus, parents and caregivers’ view should be valued as a tool to understand the environment in which young patients are raised, but with attention to a possible disagreement with the view of the patients themselves.

The exploratory subgroup analysis aimed to investigate two hypotheses: 1) the effect of CAH and treatment on the HRQoL of girls compared to boys have a greater impact due to the effects of virilization of girls and 2) patients with more severe clinical presentations (SW) experience greater losses. To both questions the answers were inconclusive.

Most studies in patients with CAH were performed in women, making outcomes in male patients much less known. Recently, a study performed in male patients with CAH showed that their HRQoL is similar to healthy patients. Interestingly, overtreatment with GC showed worse results in the HRQoL of these patients then undertreatment, reassuring the importance of adequate androgen levels [31].

We did not detect changes in HRQoL when comparing individuals by sex of rearing. It is possible that the hyperandrogenism and genital ambiguity, which we assumed to impact only girls, do not exert alone a negative influence on HRQoL of patients with CAH. The biggest impact would be just the fact of having to deal with a chronic illness requiring ongoing care, affecting the sexes equally.

Surprisingly, our patients with gender dysphoria were not outliers. However, recent study on HRQoL of DSD patients showed that girls with CAH who experienced gender dysphoria had lower scores compared to the study group at large, meaning worse HRQoL associated with gender identity disorders [32].

Regarding the clinical presentations of CAH, there was no significant difference between the mean scores of HRQoL of SW versus SV, except in the physical dimension of the CHQ. Surprisingly the most affected, for parents, were the SV. In a previous study, Frisén and colleagues have distinct findings [21], correlating the impact on HRQoL with patients with severe genotype forms of CAH.

Clinical severity of disease, as well as in our study, often is not related to individual perceptions, which explains the importance of an environment that offers help and support as a strategy for improving HRQoL [2], as well as an effective healthcare system, able to promptly diagnose and provide specialized care to patients with CAH.

Our study, like many others, had a weak sample size. Unfortunetely, we were not able to evaluate the influence on HRQoL of different treatment choices, adherence to therapy or timing of the diagnosis of CAH. Furthermore, the use of multiple generic tools for assessing HRQoL in various papers published and the absence of a validated CAH specific instrument makes it difficult to extrapolate the results. It is possible that some of the questions we want to answer would be best understood with qualitative studies on the topic.

## Conclusions

We found a seemingly impairment in the major domains of HRQoL in children and adolescents with CAH compared to healthy Brazilian children and adolescents. The self-assessment was concordant in key areas with the assessment made by their parents or guardians. There seem to be no differences in HRQoL between genders and clinical presentation forms of the disease.

## Abbreviations

CAH: Congenital adrenal hyperplasia
HRQoL: Health related quality of life
GC: Glucocorticoid
ACTH: Adrenocorticotropic hormone
SW: Salt wasting
SV: Simple virilizing
DSD: Disorders of sex development
QoL: Quality of life
SD: Standard deviation
CHQ: Child health questionnaire – PF50
PedsQoL: Pediatric quality of life inventory 4.0
